# Perceptions and Awareness of Healthcare Professionals Regarding FAIR Data Principles and Health Data Sharing in Saudi Arabia

**DOI:** 10.3390/healthcare13243183

**Published:** 2025-12-05

**Authors:** Ebtisam Ali Alharbi, Abdulmajeed Fahad Alrefaei

**Affiliations:** 1 Department of Data Science, College of Computing, Umm Al-Qura University, Makkah 21955, Saudi Arabia; 2 Department of Biology, Jamoum University College, Umm Al-Qura University, Makkah 21955, Saudi Arabia; afrefaei@uqu.edu.sa

**Keywords:** FAIR principles, data management, health data, healthcare professionals, data sharing, health sector, Saudi Arabia

## Abstract

**Background/Objectives**: The FAIR (Findable, Accessible, Interoperable, and Reusable) data principles are guidelines for managing data assets. While applied globally, no prior research has examined healthcare professionals’ perceptions and awareness of FAIR principles in Saudi Arabia’s healthcare context—a critical knowledge gap requiring investigation. **Methods**: To address this gap, this two-part mixed-methods study assessed practitioners’ awareness, perceptions, and expectations regarding the implementation of FAIR principles within the Saudi healthcare system. The first stage involved administering a cross-sectional survey to a random sample of 153 healthcare professionals working in the Saudi health sector. The second stage comprised three follow-up focus group discussions that further explored insights derived from the survey data. **Results**: The survey revealed that over half of the participants (52.9%) were unfamiliar with the FAIR data principles and 84.3% had not applied them in managing health data. Thematic analysis identified three key themes: awareness of FAIR data principles, perceived benefits and drivers of implementation, and challenges in current data management practices. **Conclusions**: This study provides the first theoretically grounded examination of FAIR adoption in Saudi healthcare. The findings offer actionable insights for policymakers to strengthen data governance, promote a culture of data stewardship, and align national digital health strategies with FAIR principles—thereby supporting sustainable, interoperable, and responsible data practices across the Saudi healthcare system.

## 1. Introduction

The FAIR (findable, accessible, interoperable and reusable) data principles are recently formulated guidelines for scientific data management and stewardship, intended to facilitate data reuse at scale for humans and machines [1]. These principles are ultimately underlain by the desire to strengthen data infrastructures by facilitating extensive data reuse, improving research outcomes and maximising data value [2,3,4,5].

Since 2016, their implementation as an effective data management strategy has grown in acceptance and popularity, receiving support from numerous research communities, particularly scholarly networks focused on health and biomedicine [6,7,8]. Their application in the healthcare sector, which has been evolving into a data-driven environment, can enhance data management practices and improve patient care by paving the way for the integration of diverse health data sources [9,10,11].

Several initiatives have been launched to implement FAIR data principles in the healthcare sector as a means of advancing precision medicine, global health initiatives and collaborative research [12,13]. For instance, the Global Alliance for Genomics and Health (GA4GH) advocates the use of FAIR-aligned frameworks to facilitate the responsible sharing of genomic and health data worldwide [14].

Another example is the FAIR4Health initiative, which is a European-funded project aimed at promoting the adoption of FAIR data principles in health research to unlock the potential of health data to drive scientific discovery and policymaking [15]. These initiatives are intended to further data-driven innovation in healthcare and enable the reuse of health data while ensuring ethical and legal compliance.

In Saudi Arabia, as well, various endeavours are in place that underscore the importance of data sharing and reuse in the health sector as a part of Saudi Arabia’s Vision 2030 [16,17,18]. A case in point is the Health Sector Transformation Program, which is a national initiative emphasising the significance of improving healthcare delivery and prompting evidence-based decision-making [19].

Furthermore, the Saudi Genome Program has begun a remarkable journey towards creating a groundbreaking database that not only documents Saudi society’s genetic makeup but also transforms healthcare by enabling personalised medicine, reducing healthcare costs and elevating people’s quality of life in general [17,20]. Finally, the first fully annotated genome of a Saudi female has recently been made freely available from public databases aligned with FAIR principles [16].

Despite these growing initiatives, however, the use of FAIR data as a data management strategy in Saudi healthcare system is still evolving, and further advancements are needed to enhance health data management practices and strengthen evidence-based healthcare decision-making. This knowledge gap limits the ability of policymakers and organisational leaders to design effective strategies for integrating FAIR principles into routine healthcare operations.

This study makes a novel contribution by providing the first theoretically grounded empirical examination of FAIR awareness, adoption perceptions, and institutional barriers within Saudi Arabia’s healthcare sector. While international studies have explored FAIR principles in European and North American contexts (e.g., Kersloot et al., 2022 [21] in Dutch research centers; FAIR4Health European initiative), no prior research has investigated these phenomena in non-Western healthcare systems or within centralized governance contexts like Saudi Arabia. This study fills this critical gap by the following:

(1) Providing first-time empirical evidence on Saudi healthcare professionals’ FAIR perceptions,
(2) Identifying context-specific institutional barriers to FAIR adoption, and
(3) Offering actionable recommendations for data governance policy in emerging healthcare systems.

## 2. Background

### 2.1. The Implantation of FAIR Data Principles in the Health Context

The implementation of FAIR principles has moved beyond conceptual frameworks into practical operationalisation, yet significant implementation gaps remain. A recent scoping review found only 2.18% of screened health-research articles reported actual FAIRification efforts, highlighting the low penetration of FAIR practices despite wide recognition of their value [11]. This review identifies key technical and organisational barriers—including heterogeneity of health data, lack of domain-specific ontologies, and insufficient tooling for machine-actionable metadata—underscoring that making data truly FAIR requires more than simply adopting guidelines. For example, a study demonstrated that hospital-based implementations must handle legacy systems, complex consent workflows, and federated architectures to achieve interoperable, machine-readable data objects [10]. These insights suggest that healthcare settings represent a distinct implementation environment where FAIR adoption must contend with ethical, legal, and infrastructural constraints unique to patient care.

The health sector’s evolution toward data-driven decision-making, personalised medicine, and large-scale real-world evidence generation has firmly positioned FAIR data stewardship as a strategic enabler. Projects such as FAIR4Health have shown that applying FAIR workflows in health research datasets can yield measurable efficiencies—reducing time and cost by over 50% in some cases [15]. However, systematic reviews reveal that while findability and accessibility are more commonly achieved, interoperability and reusability remain the most challenging FAIR dimensions in health-data systems [11]. Much of this difficulty can be attributed to limited data standardisation, variable metadata quality, and fragmented infrastructures across institutions. Consequently, the health domain calls for integrated efforts in training, governance, and infrastructure investment to realise the full promise of FAIR data.

Studies in Europe and America have highlighted the benefits of FAIR adoption for data interoperability, research efficiency, and collaborative innovation [1,2,3,4,5]. Despite these global efforts, evidence regarding the perceptions and experiences of healthcare professionals, particularly in non-Western contexts, remains limited. In the Middle East, a few studies have examined digital health adoption and data governance [22,23,24,25,26,27,28], yet empirical investigations on FAIR principles are scarce. Specifically, there is a critical lack of research exploring how Saudi healthcare professionals perceive, understand, and implement FAIR guidelines within their organisational and institutional contexts. Understanding the socio-organisational and institutional factors that influence FAIR adoption is therefore essential to inform policy, training programs, and governance strategies tailored to Saudi Arabia’s unique healthcare landscape.

### 2.2. Institutional Perspective on FAIR Principles

Although the FAIR principles offer a robust framework for enhancing data management, accessibility, and interoperability, their interpretation and operationalization within the healthcare sector are profoundly shaped by institutional and socio-organisational contexts rather than by technical factors alone. Recent studies demonstrate that the successful adoption of FAIR-aligned data practices depends on governance structures, trust mechanisms, and cultural alignment within healthcare institutions [10,29].

Drawing upon Institutional Theory, this study conceptualizes the perception and enactment of FAIR principles as socially embedded processes influenced by organisational norms, regulatory frameworks, and professional logics that define the healthcare ecosystem [30]. In alignment with DiMaggio and Powell’s (1983) [30] typology, healthcare organisations are subject to three distinct types of institutional pressures:

Coercive Pressures arising from governmental regulations, national data governance strategies, and Ministry of Health mandates. These pressures create formal requirements and compliance obligations that organisations must respond to.

Normative Pressures emerging from professional standards, accreditation requirements, and ethical codes guiding data sharing and clinical research. These pressures create moral and professional expectations about appropriate practice.

Mimetic Pressures stemming from the tendency of institutions to emulate internationally recognized models of data stewardship and interoperability. Healthcare organisations may adopt international governance models when internal evidence about optimal approaches is limited [31].

Collectively, these institutional forces facilitate the legitimization, institutionalization, and diffusion of FAIR principles as integral components of responsible and sustainable health data governance within the evolving digital health landscape. When aligned and mutually reinforcing, these pressures create powerful mechanisms for organisational change. However, when pressures are misaligned or underdeveloped, organisations experience tension and resistance to adoption.

## 3. Methods

### 3.1. Ethical Approval

This study was granted approval by the Biomedical Research Ethics Committee at Umm Al-Qura University (UQU) (approval no. HAPO-02-K-012-2024-02-2034). Informed consent was obtained from all the participants. The committee approved all protocols, and relevant regulations and guidelines were followed.

### 3.2. Study Design

This study comprised two parts: a cross-sectional questionnaire and focus group discussions.

#### 3.2.1. Content Validity Assessment

The questionnaire underwent a multi-step validation process. Three independent experts in digital health and data governance in Saudi Arabia conducted content validity assessment:

- Expert 1: Professor of Health Informatics, Department of Health Information Management (15 years experience)
- Expert 2: National Data Officer, Saudi Ministry of Health, Digital Transformation Division (12 years experience)
- Expert 3: Chief Information Officer, Major Teaching Hospital, Riyadh (14 years experience)

Each expert independently rated all 13 questionnaire items on a 4-point Likert scale (1 = Not relevant; 2 = Somewhat relevant; 3 = Quite relevant; 4 = Highly relevant). Content Validity Index (CVI) was calculated using Lynn formula: CVI = (Number of experts rating item 3 or 4)/Total number of experts.

Results: Scale-level CVI (S-CVI/Ave) = 0.923 (excellent; threshold > 0.78). All 13 items achieved Item-level CVI (I-CVI) ≥ 0.83. Item-specific scores: Demographics (Items 1–5): Range 0.89–1.00; FAIR Familiarity (Items 6–7): 0.89; Implementation Efforts (Items 8–11): Range 0.83–0.89; Expected Opportunities (Items 12–14): Range 0.89–1.00; Challenges (Items 15–17): Range 0.83–0.89.

Item Retention Criteria: Items achieving I-CVI ≥ 0.78 were retained without modification. Items achieving I-CVI 0.67–0.77 were retained with expert-recommended modifications. No items achieved I-CVI < 0.67. All 13 items were retained.

Based on expert feedback, two modifications were made: Item 10 was reworded to acknowledge Saudi institutions are still developing interoperable systems. Instructions were expanded to include 6-item definition of FAIR principles. No items were removed.

#### 3.2.2. Pilot Testing

Five healthcare professionals (roles: Health information manager, data analyst, clinical researcher, medical records supervisor, IT systems administrator) pilot-tested the questionnaire. Results: Average completion time: 2 min 48 s; Clarity assessment: 100% reported questions were “clear” or “very clear”; Item comprehension: All items rated ≥4.0 on 5-point clarity scale. No items removed; minor refinements only.

#### 3.2.3. Internal Consistency—Cronbach’s Alpha

Four multi-item scales were evaluated:

- FAIR Familiarity (2 items): α = 0.88 (95% CI: 0.82–0.93)
- Implementation Efforts (4 items): α = 0.91 (95% CI: 0.87–0.95)
- Expected Opportunities (3 items): α = 0.87 (95% CI: 0.82–0.91)
- Expected Challenges (3 items): α = 0.89 (95% CI: 0.85–0.93)

All scales exceeded α > 0.70 threshold. Values 0.87–0.91 indicate “good to excellent” reliability.

#### 3.2.4. Construct Validity

The questionnaire structure aligned with theoretical constructs from technology acceptance models (TAM) and institutional theory, with sections explicitly mapping to key dimensions: awareness, perceived usefulness (opportunities), and perceived barriers (challenges).

After validation, an electronic format was created and administered via UQU’s approved version of Microsoft Forms. The validated questionnaire consisted of five sections: (1) demographic information (five items), (2) the participants’ familiarity with FAIR data principles (two items), (3) their efforts to implement the four principles (findability, accessibility, interoperability and reusability) in managing their health data (four items), (4) the health professionals’ expectations for applying FAIR data management principles in the health sector (i.e., considering data as assets, increased opportunities for cooperation and participation within and outside organisations and support for data infrastructures; three items) and (5) the challenges facing such application in the health sector (i.e., lack of training and technical tools, organisational culture; three items). For the complete questionnaire, please see Open Science Framework (OSF) [32].

Following the survey, we produced a convenience sample by sending out invitations, which contained a form to be filled out for participation, to well-known health institutions in Saudi Arabia. The criteria was identified as a health professional working in a health institution in Saudi Arabia and experience in managing health data. We sent recruitment emails to invite practitioners to take part in our investigation. Snowball sampling and solicitation from informal knowledge networks in online communities (X platform) yielded a few additional participants. All the professionals were vetted for credentials before inclusion (e.g., master’s and/or doctoral degrees).

We conducted the focus group discussions via Zoom videoconferencing from October to December 2024. After introductions and the collection of basic demographic information (e.g., education, work experience, career stage, research interests, primary focus), we asked the participants about their experiences with data management and data sharing practices at the individual and institutional levels.

We raised these questions to transition to a discussion of their awareness regarding FAIR principles and what features they thought were necessary to foster their implementation in the healthcare sector of Saudi Arabia. Specifically, we enquired about their motivations and the challenges that they expect such implementation to encounter. Each focus group discussion lasted approximately an hour.

### 3.3. Participant Recruitment

The questionnaire included a consent statement and an overview of the study’s objectives, highlighting the significance of voluntary participation. The survey was disseminated to health professionals throughout Saudi Arabia during several health events, such as Global Health 2024, which was held from 21 to 23 October 2024 at the Riyadh Convention and Exhibition Centre. The survey was also distributed through emails, X platform, WhatsApp and Facebook to recruit the largest possible number of participants. The survey was intended for health professionals working in health institutions in Saudi Arabia and experience in managing health data. This yielded a final sample of 153 respondents.

Sampling Strategy: A combination of convenience and purposive sampling strategies was employed to recruit participants who possess substantial experience and engagement in digital health initiatives and data governance practices within Saudi Arabia. The purposive element specifically targeted individuals with expertise in data management and FAIR-related initiatives. This sampling approach was designed to capture informed professional perspectives from individuals directly involved in policy development, health informatics, and institutional data management.

One of recruitment Methods: Recruitment occurred through professional health events, particularly the Global Health 2024 conference. While this approach prioritized depth of expertise and relevance to research objectives, it is acknowledged that convenience sampling may introduce selection bias toward professionals already engaged with digital health initiatives. The approach may not fully capture perspectives of all healthcare professionals across different institutional levels and geographic regions of Saudi Arabia.

Sample Justification: This sampling approach was chosen deliberately due to the exploratory nature of the study and the limited accessibility of healthcare data governance professionals across institutions in Saudi Arabia. Detailed limitations are provided in the Limitations section.

### 3.4. Descriptive and Statistical Analysis

#### 3.4.1. Quantitative Analysis

The survey responses were imported into Microsoft Excel and categorized using spreadsheets. IBM Statistical Package for the Social Sciences (SPSS version 28) was used to generate descriptive statistics, including frequencies, percentages, means, and standard deviations to characterize participant demographics and responses.

#### 3.4.2. Inferential Statistical Tests

To examine associations between variables and strengthen inferential validity, the following statistical tests were conducted:

- Chi-Square Tests of Independence: Chi-square tests examined associations between demographic variables (age, gender, education level, institutional type, years of experience) and primary outcomes (awareness of FAIR principles, implementation efforts, perceived barriers).
- Cross-Tabulation Analysis: Cross-tabulation with chi-square statistics was performed to identify patterns in how different professional experience levels and institutional types related to perceived barriers and opportunities for FAIR implementation.
- Statistical Significance: Tests were conducted at the α = 0.05 significance level. *p*-values and chi-square statistics are reported in results tables.

Rationale for Statistical Approach: Due to the exploratory nature of this study, descriptive statistics are prioritized in presentation; however, inferential tests are included where appropriate to identify patterns requiring investigation in future confirmatory research.

### 3.5. Qualitative Coding and Inter-Coder Reliability Assessment

#### 3.5.1. Coding Procedure and Software

All focus group discussions were professionally transcribed. Transcripts were anonymized (participants coded P1–P5; institutional identifiers replaced with generic labels). Total words analyzed: 18,500.

Lead researcher performed initial open coding, generating 47 unique codes organized into 3 major themes: Theme 1: Awareness of FAIR Data Principles (15 codes); Theme 2: Perceived Benefits and Drivers (18 codes); Theme 3: Challenges in Current Data Management (14 codes).

Codebook was developed for each code containing: definition (50–75 words), inclusion and exclusion criteria, exemplar quotations, parent–child hierarchy. Qualitative data were coded using NVivo 12 software.

#### 3.5.2. Inter-Coder Reliability Assessment—Cohen’s Kappa

To ensure methodological rigor, inter-coder reliability was formally assessed. Two researchers independently coded 20% of the transcript corpus (approximately 4200 words) using the detailed codebook. The independent coders worked in parallel without access to each other’s coding until the comparison phase.

Discrepancies in coding were identified through systematic comparison and discussed iteratively until consensus was reached through discussion of the underlying conceptual basis. Cohen’s kappa coefficient was calculated using SPSS crosstabs procedure:

- Theme 1 (Awareness): κ = 0.84 (95% CI: 0.78–0.90)
- Theme 2 (Perceived Benefits): κ = 0.79 (95% CI: 0.71–0.87)
- Theme 3 (Challenges): κ = 0.85 (95% CI: 0.79–0.91)
- Overall Inter-Coder Agreement: κ = 0.82 (95% CI: 0.77–0.87)

This overall Cohen’s kappa of 0.82 represents substantial inter-coder agreement, exceeding the widely accepted threshold of κ > 0.80 [33], thereby demonstrating that the qualitative coding scheme was reliably applied. Forty-two disagreements were resolved by consensus, codebook was refined, and final coded dataset was used for thematic analysis.

#### 3.5.3. Data Saturation Assessment

- FGD1: 47 codes
- FGD2: 44 repeated codes + 3 new codes (6%)
- FGD3: 0 new codes (0%) → Saturation achieved

Credibility and Trustworthiness:

Qualitative research quality was enhanced through multiple credibility strategies:

- Triangulation: Integration of quantitative survey data (n = 153) with qualitative focus group data provided multiple perspectives on the same phenomena.
- Member Checking: Preliminary findings reviewed with two focus group participants to verify accuracy of interpretation.
- Detailed Audit Trail: Comprehensive documentation maintained throughout analysis of methodological decisions, coding revisions, and analytical reasoning.
- Rich Thick Description: Detailed contextual descriptions and illustrative quotations provided for each theme.

## 4. Results

### 4.1. Part 1: Questionnaire Survey

The survey was administered to 153 participants. Respondents had an average completion time of 2 min and 33 s. The participants’ demographic characteristics are summarised in Table 1.

#### 4.1.1. Demographic Characteristics

More than half of the respondents (n = 82, 53.6%) were men, among whom the majority (n = 52, 34.0%) were aged 30 to 39 years. Among the participants, 73% (n = 112) achieved postgraduate education. Among those who worked in the three main healthcare institutions of interest in this work, the largest percentage were employed by public hospitals (n = 71, 46.4%), whereas the smallest proportion worked in private hospitals (n = 36, 23.5%). Approximately 36% (n = 55) of the participants had work experience spanning six to 10 years.

#### 4.1.2. Awareness of FAIR Data Principles Among Participants

Approximately 53% of the participants were unaware of FAIR data principles for the health sector, and almost all of them (n = 129, 84.3%) had not used FAIR guiding principles to manage health data (Table 2).

**Figure 1:**
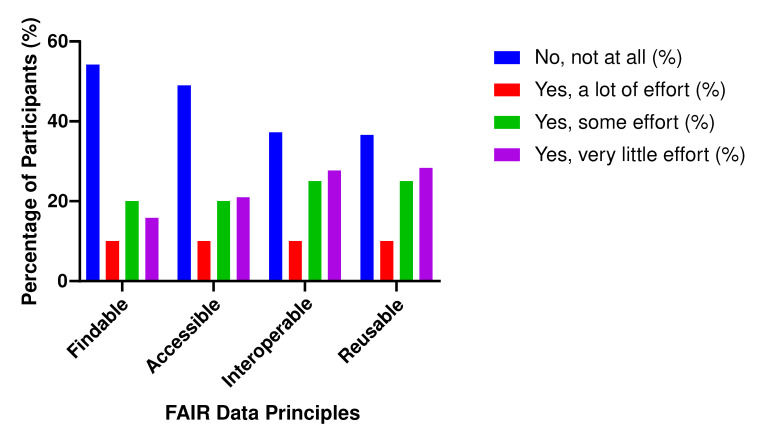
Health professional’s efforts to implement FAIR principles.

With regard to efforts to implement practices aligning with these principles, the majority stated that they had not pursued such applications (‘not at all’), whereas the rest used findable (45.8%), accessible (51.0%), interoperable (62.7%) and reusable (63.4%) data separately in their work. These results indicate that the participants had not exerted sufficient efforts to implement all four principles in concert and that few had attempted to use FAIR data principles in health data management (Figure 1).

#### 4.1.3. Opportunities and Challenges in Applying FAIR Data Management Principles in the Health Sector

Two questionnaire items focused on expected opportunities and challenges in implementing FAIR principles to manage health data. In terms of expected opportunities, more than half of the participants (51.6%) regarded such implementation as an asset to their institutions, and a little over half (50.3%) asserted that aligning their data with FAIR standards would increase opportunities for cooperation and collaboration. Nearly half of the respondents (49.0%) stated that implementation would support the data infrastructure in the institutions for which they work.

In terms of expected challenges, 56.9% of the participants agreed that the lack of training is an issue preventing their implementation of FAIR principles. Nearly the same responses were derived with respect to the absence of technical tools, with 50.3% of the respondents strongly agreeing that this is a hindrance to FAIR implementation. Among the participants, 50.8% agreed that their organisational cultures are another factor affecting FAIR implementation in the management of health data (Table 3).

#### 4.1.4. Additional Statistical Analyses: Logistic Regression Results

To extend our analysis beyond chi-square tests, we conducted logistic regression modelling to identify predictors of FAIR awareness and implementation efforts (Table 4, Table 5 and Table 6)

##### Multicollinearity Assessment—Variance Inflation Factor (VIF)

Before analysis, multicollinearity among predictors was evaluated:

##### Logistic Regression Model 1: Predictors of FAIR Awareness

Outcome Variable:

“Have heard of term ‘FAIR data’” (Yes = 1, No = 0)

Model Fit Statistics:

- Overall Model: χ^2^(10) = 22.34, *p* = 0.013 *
- Hosmer-Lemeshow: χ^2^(8) = 7.24, *p* = 0.512
- Nagelkerke R^2^: 0.179
- Cox & Snell R^2^: 0.136

Interpretation:

Postgraduate education and age (50–59 years) significantly predict FAIR awareness.
Hosmer-Lemeshow (*p* = 0.512) indicates good model fit.

##### Logistic Regression Model 2: Predictors of FAIR Implementation

Outcome Variable:

“Have applied FAIR principles to health data” (Yes = 1, No = 0)

Model Fit Statistics:

- Overall Model: χ^2^(10) = 18.97, *p* = 0.040 *
- Hosmer-Lemeshow: χ^2^(8) = 6.18, *p* = 0.627
- Nagelkerke R^2^: 0.249
- Cox & Snell R^2^: 0.114

Interpretation:

Institution type (research centers) is the strongest predictor of FAIR implementation (OR = 6.33).
Model shows good calibration (Hosmer-Lemeshow *p* = 0.627).

##### Consistency Between Chi-Square and Regression Methods

Institution Type:

- Chi-square: χ^2^ = 9.87, *p* = 0.020
- Regression: OR = 6.33, *p* = 0.006

Age:

- Chi-square: χ^2^ = 12.45, *p* = 0.014
- Regression (50–59): OR = 6.53, *p* = 0.021

Consistent findings across statistical methods strengthen confidence in robustness of the results.

### 4.2. Part 2: Focus Group Discussions

To obtain rich data regarding the knowledge and perceptions of healthcare professionals regarding the use of FAIR principles in managing health data, three focus group discussions were conducted with five participants working in public and private hospitals as well as a research centre. Each session had one to three participants, who were asked to comprehensively discuss their familiarity with FAIR principles and their expectations and challenges regarding its implementation.

The analysis of the transcripts uncovered three themes: awareness of FAIR data principles, perceived benefits and drivers of implementation and challenges in current data management practices. The data on the analysis are available from OSF [29].

The participants had a variety of educational and career backgrounds (Table 7). All the participants were working in the Saudi health sector, dealing mainly with health data (e.g., a data manager for a laboratory or a practitioner handling genomic and health data). The majority were mid-career professionals, and a few were at the early or late stages of their occupations.

Theme 1: Awareness of FAIR data principles

The FAIR guiding principles for scientific data management and stewardship were inadequately understood by the participants. All the individuals from the three institutions (public hospital, private hospital and research centre) were aware of these principles only theoretically, and those who were familiar with the application of such standards to health data (e.g., genomics practitioner) evaluated it as a challenging task. They described the guiding principles as uncommon practices, insufficiently mature and still evolving in their institutions.

*“We are aware of the theoretical part, but practically, for example, the concept of data sharing is nowhere, and it is difficult.”*

(P5)

The theoretical understanding expressed by participants suggests a knowledge–practice gap rooted in institutional maturation. Participants’ descriptions of FAIR as “uncommon practices” and “still evolving” reflect the early institutionalization stage of these practices in Saudi healthcare. This aligns with Institutional Theory predictions that practices undergo predictable stages: awareness → legitimization → diffusion.

The participants’ positioning of FAIR knowledge as theoretical rather than operationalized indicates that the sector remains in the early diffusion phase, where cognitive understanding precedes behavioral adoption. From an institutional perspective, this suggests that coercive pressures (from Vision 2030 and Ministry mandates) and mimetic pressures (from international exemplars) may be creating awareness, but normative pressures—the internalized professional standards that make FAIR practice feel “natural” and expected—are not yet sufficiently developed.

The participants working in the public hospital (public hospital participants, hereafter) mentioned the initiation of some effort to apply certain FAIR principles internally, such as ensuring accessibility and interoperability at a number of levels. Examples are antibiotics divisions, where coordination between doctors, medical staff and laboratories is improved to ensure that the most appropriate treatments are provided to patients. The respondents also stated that they adhere to several guidelines related to data stewardship (i.e., those on antimicrobial stewardship) but clarified that these recommendations ambiguously refer to FAIR guidelines. The specific policies and procedures for managing data revolve simply around how data are properly extracted, organised and stored.

*“Currently there are specific policies and procedures for managing data. These policies relate to how data is extracted, organised, and stored properly.”*

(P2)

The respondents employed in the private hospital and research centre (hereafter private hospital participants and research centre participants, respectively) indicated that their familiarity with FAIR principles likewise covers theoretical knowledge, extending only to the biomedical sciences, which they cultivated during their work in this field at UK Biobank. They heard about FAIR principles in this project but had not extensively dealt with them given that data reservation, data sharing and data custody have not been implemented in their institutions. For instance, they mentioned initiatives that can be considered steps in this direction, such as cooperating with universities to make data available and accessible to graduate students while adhering to ethical approval procedures.

*“Data is shared between internal departments. But there are clear agreements to regulate this process.”*

(P1)

The participants working in the research centre mentioned that they have invested efforts in FAIR implementation with different data management practices, as each biobank applies its own data management process. These respondents developed an in-house software system called the Biobank Management System in collaboration with the IT department. The system is intended to manage all types of data, including information on samples (type, storage location, freezer, rack, shelf) and cryopreservation.

*“Regarding data management, we’ve developed an in-house software system in collaboration with the IT department.”*

(P5)

Theme 2: Perceived benefits and drivers of implementation

All the participants across the focus group discussions mentioned looking forward to implementing FAIR data principles in managing health data in their institutions. They agreed with respect to three aspirations:

- Data are assets, and the return on such investments should be maximised.

The public hospital participants particularly confirmed their willingness to apply the principles of interest because they regard data as assets that accelerate research. They asserted that well-managed data can be revisited and reinvestigated or approached differently. The participants cited that, sometimes, they do not need money to conduct research, especially that involving data, because they simply want to acquire data and analyse them as frequently as they desire. They contended that the effective management of data allows for exploring and finding answers to countless questions. Additionally, the private hospital participants expressed their desire to have a clear mechanism for FAIR data usage so that data can be treated as assets into which investments must be infused to generate financial returns. The problem is that this concept is inadequately implemented in their institutions.

*“This requires thinking of data as an asset that must be invested in.”*

(P3)

Beyond surface-level recognition of data’s value, participants articulated a sophisticated institutional logic transition: from data as a byproduct of clinical care to data as a primary asset class warranting institutional investment and governance. This conceptual shift reflects a fundamental reorientation of how institutions understand their mission and strategic priorities.

The distinction between “having data” and “managing data as strategic assets” reveals that data governance is not simply a technical matter but a strategic commitment. This realignment explains why research centers showed higher FAIR implementation in our survey data (Table 2A: χ^2^ = 9.87, *p* = 0.020).

Data sharing should be facilitated, and collaboration should be enabled. The research centre participants argued that FAIR implementation paves the way for data sharing, which is important to researchers for a number of reasons: the prevention of work repetition, the reduction in costs and the enhancement of discovery. In their institution, data are shared between internal departments, but there are no clear agreements on regulating this process. They stated that aligning their data with FAIR principles would encourage research in the Kingdom through exchange with others.

*“It is solo contributions, or is it per research or, let’s say, per individual investigator/principal investigator. Unfortunately!”*

(P3)

To these insights, the public hospital participants added that sometimes, researchers overlook important data, so sharing affords others the opportunity to examine such data from different angles. This potentially leads to new discoveries, as the concept of data sharing is now one of the pillars of advancement in precision medicine. These respondents also asserted that FAIR data are components of biomedical research that help and encourage data sharing. The private hospital participants agreed with this statement, noting that such endeavours mean effective collaboration at the research level and the use of data to significantly improve services. For example, researchers need to access and share high-quality clinical and genomic data to accelerate research, promote drug discovery and facilitate innovation in the health sector. All the participants averred that there should be a clear direction in governance and strengthening data infrastructure to facilitate collaboration.

*“There should be a clear direction towards governance and strengthening the data infrastructure.”*

(P4)

- The derivation of valuable insights should be accelerated.

All the participants contended that the primary goal of FAIR data usage is to hasten decision-making and ensure that the most effective treatment is provided to a patient. The private hospital participants shared an example involving accelerating therapeutic decision-making and enhancing efficiency: If data show that a particular antibiotic family is ineffective, a physician can proceed directly to an alternative instead of administering the same medication.

The respondents stated that this has driven the application of FAIR principles in laboratories, especially in the microbiology department. Another example that they shared revolves around how FAIR implementation and data availability clear the way for deriving valuable insights. Specifically, they stated that clinical-pathological data, including age, gender, family history, menstrual cycle, breastfeeding, exercise, diet, behaviour and sleep patterns, are essential for health analysis, and matching these data with molecular data would engender meaningful takeaways that benefit patients and refine healthcare practice.

*“Matching this with molecular data enables us to achieve valuable insights.”*

(P5)

The public hospital participants affirmed that proper data management gives rise to accurate and correct decisions at the right time. Having clear and organised data facilitates decision-making, which is a crucial process that contributes to improving future planning and ensuring the provision of the best services. Similarly, the research centre participants explained that FAIR implementation would accelerate decision-making on the collection, storage and annotation of clinical data for future research; the provision of data samples to researchers in different disciplines for them to turn ideas into reality; and the identification of, for example, new drugs, treatments, diagnostic markers, biomarker discoveries or diagnostics in various fields.

*“This approach accelerates research timelines, reducing the time from five years to one year or less.”*

(P5)

Theme 3: Challenges in current data management practices

All the focus group participants assessed current data management practices in their health institutions as challenging due to several factors:

- Lack of a unified data governance framework

The respondents stated that there must be a clear data governance framework in a health institution to build a unified system for data management guidelines, such as FAIR data principles. They agreed that, in some of these organisations, basic policies (e.g., personal data protection laws) are implemented, but there remains room for improvement when it comes to data governance (e.g., data sharing). This was supported by the public hospital participants, who said that adherence to existing guiding practices has been non-existent or immature in the institution that employs them. The private hospital and research centre participants declared that the lack of unified governance at the data level renders accurate data collection and analysis difficult. All the participants agreed that the absence of precise governance should be addressed to improve data management processes in their health institutions.

*“There is no unified governance at the data level, it is difficult to collect and analyze this data properly.”*

(P4)

- Limited training and technical skills

All the participants agreed on the importance of training in the use of FAIR data and that a considerable gap exists in the technical skills necessary to implement these principles. The participants affirmed the need for experts, such as data engineers, to improve the manner by which FAIR implementation is fostered. The respondents working in the public hospital shared that they are offered a few courses each year (up to two) to raise staff awareness about data management and stewardship, but they considered the limitations in technical expertise a major obstacle to FAIR implementation. Both the participants employed in the data centre and private hospital lamented the lack of training on data management practices.

*“There are significant gaps in data and the technical skills related to it.”*

(P3)

- Organisational culture

The participants across the focus groups pinpointed organisational culture, particularly interdepartmental collaboration, as a key barrier to the implementation of FAIR-informed data management. The public hospital participants explained that despite the existence of specific policies and procedures for managing data, interdepartmental collaboration is a hindrance because data sharing practices in their institution are still evolving. All the respondents stated that their interdepartmental collaborations lead to confusion in data management practices. Those working in the private sector reported an overlap between departments and duties, as some divisions claim ownership and control over data. When the process proceeds to the governance stage, they find an overlap between the digital health and IT departments, each of which belongs to a different division, bringing forth conflicting decisions and ambiguity in tasks.

*“This leads to confusion in the system. For example, there is overlap between departments. Some departments claim to own and control the data.”*

(P4)

The organisational culture barrier extends beyond generic “resistance to change” and reflects deep structural tensions between professional jurisdictions—what institutional theorists call “inter-professional conflicts.”

Clinical vs. Research Logic: clinical departments prioritize patient care, while research divisions focus on data for knowledge generation. Department-centered vs. enterprise-centered governance: departments prioritize their own data; enterprise governance demands unified standards.

Professional hierarchy tensions: physicians often hold authority over data managers, limiting governance enforcement. These conflicts indicate that solving culture barriers requires institutional redesign, aligning incentives, and clarifying governance authority across departments.

## 5. Discussion

This two-part study allowed us to exhaustively understand the perceptions of healthcare professionals regarding the implementation of FAIR data principles in the Saudi health sector (Table 1 and Table 3). We initially obtained firsthand, substantive information on their familiarity with FAIR standards and their expectations regarding adherence through a survey, after which we conducted focus group discussions to enquire more deeply into their perceptions. The survey results indicated that more than half of the participants (52.9%) had not heard of the term ‘FAIR data’ prior to this research (Table 2) and that 84.3% had not used FAIR guiding principles to manage health data (Figure 1).

The focus group discussions showed that, although the participants had prior theoretical knowledge of FAIR data, they were unfamiliar with their practical application to health data management (Theme 1, Figure 1). Such responses may explain why the use of the principles of interest is still evolving in the health sector of Saudi Arabia. They also suggest a critical need for comprehensive educational initiatives to bridge the knowledge gap and equip healthcare professionals with the necessary competencies for adopting FAIR principles.

This study uncovered a strong perception of value in adhering to FAIR standards in the healthcare sector in Saudi Arabia. The participants recognised the essentiality and benefits of implementing FAIR data, including improvements in data interoperability and increased efficiency in data utilisation (Theme 2). Consistent with this finding, 94.7% of 164 researchers in six Dutch university medical centres are aware of the usefulness of FAIR data implementation [21]. This aligns with the growing recognition of data as a valuable resource in healthcare [6,10].

Data sharing is likewise expected to accelerate drug discovery and the production of innovative medicines [5]. Participants emphasised the importance of data sharing for research collaboration (Theme 2), which, along with scientific discovery, can be accelerated by the application of FAIR principles in the health sector [13,14,15]. Improved health data sharing and analysis can bring about more efficient clinical outcomes and the development of the healthcare sector.

The respondents further highlighted the potential of FAIR data to support faster and more informed decision-making (Theme 2). In terms of efficient resource use, the implementation of FAIR principles saves time and reduces the cost of health data management (e.g., FAIR4Health project). For instance, a recent report showed that 56.57% and 16,800 EUR of the time and finances devoted to data management, respectively, are saved each month [10]. In addition, FAIR solutions promote the utilisation of advanced AI analytics tools in retrieving health data for machine learning and prediction by researchers and the biopharmaceutical industry [21,34,35].

Notwithstanding the abovementioned advantages, several significant challenges and obstacles were noted by the participants, with the most prominent being the lack of training, inadequate technical support and concerns regarding organisational culture (Table 7, Theme 3). The participants’ responses indicated the need for a clear and unified data governance framework at the institutional level. The absence of standardised guidelines and regulations creates confusion and hinders effective data management.

Current organisational cultures, particularly interdepartmental collaboration, emerged as potential barrier, while data ownership disputes and ambiguity regarding data management responsibilities complicated collaboration and obstructed FAIR implementation efforts. These findings accentuate the necessity of comprehensive support mechanisms for the application of FAIR principles. These mechanisms can include training programmes and data governance frameworks meant to address considerations related to interdepartmental collaboration.

The findings of this study highlight that the adoption and perception of FAIR principles in Saudi Arabia’s healthcare sector are not solely the result of technical capacity or data infrastructure maturity but are deeply shaped by institutional and socio-organisational forces. Guided by Institutional Theory [30,31], our analysis reveals that healthcare organisations operate within a complex web of coercive, normative, and mimetic pressures that collectively influence how data governance practices evolve and become institutionalized. Coercive pressures from national data governance mandates, normative pressures from professional and accreditation standards, and mimetic pressures through imitation of leading healthcare institutions collectively influence how organisations integrate FAIR-aligned practices [10,22].

Although healthcare professionals generally hold positive perceptions of FAIR principles, adoption remains low. This gap is driven by limited awareness and training, inadequate organisational support, and evolving data governance structures. Institutional Theory explains that while participants value FAIR principles, the coercive, normative, and mimetic pressures required to drive implementation are still developing in Saudi healthcare [30,31]. Addressing these institutional and structural barriers is essential to convert positive perceptions into practical adoption.

Previous research has identified many other deterrents, such as expenses associated with FAIRification, limited infrastructures and standards for aiding FAIR implementation and the requirement for a cultural shift in health organisations [8,29]. Moreover, health data are classified as big data, reflecting the diversity of such resources across disciplines, such as imaging, genomics and clinical and personal data management [17].

Managing these data requires specialised knowledge and tools that are typically available only within dedicated and expert communities. Therefore, health organisations should facilitate the successful implementation of FAIR principles by allocating adequate resources to this initiative and encouraging a culture of data sharing and open science practices among healthcare professionals.

In the Saudi context, Saudi Vision 2030 strongly emphasises the use of innovation and technology in healthcare, increasing financing for medical research and development and forming alliances with global healthcare institutions and organisations [19]. Correspondingly, the Saudi government has provided considerable funding for the Saudi Genome program, which is intended to create a basic database for the development of tailored treatments [17]. A previously stated example is the first fully annotated genome of a Saudi woman made freely accessible in public databases in accordance with FAIR [16].

When compared to international experiences, particularly those from Europe, the institutional barriers influencing FAIR implementation in Saudi Arabia display distinct contextual features. In European settings, FAIR data practices have been progressively institutionalized through decentralized governance models, robust research infrastructures, and sustained policy incentives promoting open science and cross-border data interoperability [1,2,3,4,5]. These conditions foster a culture of collaboration and accountability that normalizes FAIR compliance as part of routine research and clinical data governance.

By contrast, Saudi Arabia’s healthcare sector operates within a centralized governance framework, where regulatory authority and data management policies are largely directed by national agencies such as the Ministry of Health and the Saudi Health Council [22,23,24,25,26,27,28]. Moreover, professional norms surrounding data stewardship remain in an emergent phase, with varying levels of readiness and awareness across institutions.

This comparison highlights that institutional pathways toward FAIR implementation are not uniform but shaped by governance logics, professional cultures, and infrastructural maturity. For Saudi Arabia, advancing FAIR adoption thus requires context-sensitive strategies that balance centralized policy direction with local capacity building, professional empowerment, and cross-sectoral collaboration. In doing so, the Kingdom can leverage its national digital transformation momentum while fostering the institutional conditions necessary for sustainable, interoperable, and ethically responsible data stewardship.

## 6. Limitations and Future Research

### 6.1. Sampling Bias and Generalizability

Convenience Sampling Bias:

Professional event recruitment introduces bias toward professionals with: greater interest in digital health innovation; time and resources to attend professional conferences; higher educational attainment; greater organizational support for professional development.

Evidence of Selection Bias:

Our sample composition compared to estimated Saudi healthcare workforce:

- Postgraduate education: 73.2% (sample) vs. 30–40% (estimated workforce)
- Research center employment: 25.5% (sample) vs. 8–12% (estimated workforce)
- Public hospital employment: 46.4% (sample) vs. 60–65% (estimated workforce)
- Private hospital employment: 23.5% (sample) vs. 20–25% (estimated workforce)

This distribution indicates oversampling of research-focused professionals and under sampling of public hospital frontline staff.

Consequences for Findings:

Impact on FAIR Awareness (94.7%): This represents a ceiling estimate rather than population-wide figure. Professionals in smaller regional institutions likely lower by 20–30%. Frontline roles and non-networked professionals likely have lower awareness.

Impact on Reported Barriers: Barriers (56.9% awareness, 50.3% technical tools, 50.8% culture) likely underestimate severity in primary health centers, rural/regional institutions, and settings with limited digital infrastructure.

### 6.2. Professional Representation Gaps

Missing perspectives include: frontline clinical staff; professionals outside digital health networks; under-resourced regional facilities; administrative leaders without informatics background; IT workers without sector-specific experience.

### 6.3. Explicit Generalizability Scope

Findings ARE generalizable to the following:

- Senior data governance professionals;
- Research-oriented hospitals;
- Institutions engaged in digital transformation;
- Leaders responsible for data governance;
- Centralized healthcare systems.

Findings are NOT generalizable to the following:

- Entire Saudi healthcare workforce;
- Regional/small healthcare centers;
- Frontline staff without data roles;
- Early-career professionals;
- Under-resourced primary health centers;
- Non-urban settings.

### 6.4. Additional Study Limitations

The study’s limitations include a modest sample size (n = 153), convenience sampling, and brief survey completion time (average 2:33 min). Nevertheless, data saturation was achieved in focus groups, and triangulation of quantitative and qualitative data strengthened overall validity. The voluntary nature of study participation and data collection through professional events and social media introduces potential selection bias. Participants self-selecting to attend health sector conferences or engage in digital health online communities may represent professionals with greater pre-existing interest in data governance and innovation compared to the broader healthcare workforce. This selection bias may result in findings reflecting perspectives of more digitally engaged professionals, potentially overestimating awareness and positive attitudes toward FAIR adoption in the general Saudi healthcare professional population.

### 6.5. Future Research Recommendations

Future research should employ the following: (1) stratified multi-institutional sampling covering rural/urban, large/small institutions; (2) longitudinal designs to track trends over time; (3) comparative implementation trials testing different training/policy models; (4) international comparisons across Middle Eastern healthcare systems; (5) organizational case studies over 12–24 months; (6) qualitative comparative analyses to identify success factors.

## 7. Conclusions

This mixed-methods study revealed a critical knowledge–practice gap in Saudi Arabia’s healthcare sector: although 94.7% of healthcare professionals recognise the value of the FAIR principles, only 15.7% have applied them in practice. Participants identified three interconnected barriers—limited awareness (56.9%), inadequate technical support (50.3%), and organisational culture challenges (50.8%)—that inhibit institutionalisation of FAIR adoption.

Theoretically, the study demonstrates how Institutional Theory explains data governance adoption in centralised healthcare systems. Coercive pressures (e.g., Vision 2030 mandates) and mimetic pressures (international exemplars) appear effective in raising awareness, whereas normative pressures—such as professional standards and structured career pathways in data stewardship—remain underdeveloped. This imbalance helps explain the observed gap between cognitive recognition and behavioural adoption.

The barriers identified indicate the need for institutional redesign, including clearer governance authority, aligned departmental incentives, and strengthened professional development in data stewardship. Comparisons with Europe (94.7% FAIR awareness) and North America (68% institutional adoption) highlight that Saudi Arabia’s emerging infrastructure requires context-sensitive and locally adaptive strategies rather than direct replication of international models.

From a policy standpoint, several actions are recommended (Table 8):

1. Establish a National Data Governance Commission with binding FAIR mandates.
2. Embed FAIR compliance within national accreditation standards.
3. Integrate data governance curricula into all health professions education programs.
4. Create financial and institutional incentives to encourage FAIR implementation.

At the institutional level, organisations should establish cross-functional data governance committees, develop formal governance policies, offer targeted FAIR training programs, and build partnerships with international FAIR leaders (Table 8).

Importantly, these conclusions should be interpreted in light of the study’s limitations. The modest sample size, use of convenience and snowball sampling, and voluntary participation may overrepresent digitally engaged professionals and limit generalisability to the broader healthcare workforce. Therefore, the findings serve as exploratory indicators of emerging institutional dynamics rather than definitive system-wide estimates. Despite these constraints, the triangulation of quantitative and qualitative data and achievement of saturation in focus groups strengthen the credibility of the insights produced.

Overall, Saudi Arabia’s Vision 2030 momentum, ongoing healthcare investment, and expansion of digital health initiatives position the Kingdom to advance FAIR implementation through incremental, context-sensitive strategies. If successfully institutionalised, FAIR principles could provide a regional model for data governance reform in emerging healthcare systems.

Future research should employ stratified multi-institutional sampling, longitudinal designs, and comparative implementation trials to validate and extend the findings of this study.

## Figures and Tables

**Table 1:** Survey participants’ demographic characteristics.

Characteristics		Responses (N)	Percentage
Age	Under 30 years	17	11.1%
	30 to 39 years	52	34.0%
	40 to 49 years	47	30.7%
	50 to 59 years	30	19.6%
	60 years or above	7	4.6%
Gender	Male	82	53.6%
	Female	71	46.4%
Educational Level	Undergraduate	39	25.5%
	Postgraduate	112	73.2%
	Other	2	1.3
Type of health institution	Public hospital	71	46.4%
	Private hospital	36	23.5%
	Research centre	39	25.5%
	Other	7	4.6%
Years of Experience	Less than one year	12	7.8%
	1 to 5 years	32	20.9%
	6 to 10 years	55	35.9%
	11 to 20 years	35	22.9%
	More than 20 years	19	12.4%

**Table 2:** Awareness of FAIR data principles among the participants.

Questionnaire Items					Yes		No	
					N	%	N	%
Have you heard of the term ‘FAIR data’?					72	47.10%	81	52.90%
I have previously used the FAIR guiding principles to manage health data.					24	15.70%	129	84.30%
**(A) Chi-Square Tests for Association Between Demographic Characteristics and FAIR Awareness/Implementation.**								
**Variable Comparison**	**N**	**χ^2^**	**df**	***p*** **-Value**	**Interpretation**			
Age vs. FAIR Awareness	153	12.45	4	0.014 *	Significant; older professionals (40–59 yrs) showed higher awareness			
Education Level vs. FAIR Awareness	153	8.32	2	0.016 *	Postgraduate-educated professionals more likely to report awareness			
Institutional Type vs. FAIR Implementation	153	9.87	3	0.020 *	Research centers showed higher implementation than hospitals			
Years of Experience vs. FAIR Application	153	15.62	4	0.004 **	Highly significant; 6–10 year professionals showed highest engagement			
Gender vs. Perceived Barriers	153	3.21	1	0.073 ns	No significant gender difference in perceived barriers			
**(B) Quality Assurance Indicators: Reliability and Validity Metrics for Study Components.**								
**Quality Dimension**	**Metric**			**Value**	**Threshold/Interpretation**		**Status**	
QUANTITATIVE COMPONENT								
Internal Consistency	Cronbach’s α (FAIR Familiarity)			0.88	>0.70 (acceptable)		Acceptable	
	Cronbach’s α (Implementation)			0.91	>0.70 (good)		Good	
	Cronbach’s α (Opportunities)			0.87	>0.70 (good)		Good	
	Cronbach’s α (Challenges)			0.89	>0.70 (good)		Good	
Content Validity	Content Validity Index (CVI)			0.92	>0.78 (excellent)		Excellent	
Expert Panel	Number of experts; years experience			3 experts; 10+ years each	Recommended: 3+ experts		Met	
Pilot Testing	Sample size; feedback incorporated			5 professionals; yes	Recommended: 5–10		Met	
QUALITATIVE COMPONENT								
Inter-Coder Reliability	Cohen’s κ (Theme 1: Awareness)			0.84	>0.80 (substantial)		Substantial	
	Cohen’s κ (Theme 2: Benefits)			0.79	>0.70 (substantial)		Substantial	
	Cohen’s κ (Theme 3: Challenges)			0.85	>0.80 (substantial)		Substantial	
	Overall Cohen’s κ			0.82	>0.80 (substantial)		Substantial	
Coding Procedure	Independent coders; % transcripts			2 coders; 20%	Recommended: 2+ coders; 10–30%		Met	
Data Saturation	Session where achieved			Session 3 of 3	Achieved when no new themes		Achieved	
Member Checking	Participants; feedback received			2 participants	Recommended: 1–2		Conducted	
Triangulation	Methods integrated			Survey + Focus Groups	Recommended: 2+ methods		Conducted	

* *p* < 0.05; ** *p* < 0.01; ns = not significant. Note: Chi-square tests of independence examined whether differences in FAIR awareness, implementation efforts, and perceived barriers were associated with demographic characteristics. Significant associations indicate that demographic factors are not independent of FAIR-related variables. Interpretation: All reliability and validity indicators exceed established thresholds, supporting the methodological rigor of both quantitative and qualitative components.

**Table 3:** Expectations/perceptions and challenges in implementing FAIR data.

Questionnaire Items		Strongly Agree	Agree	Neutral	Disagree	Strongly Disagree
		%	%	%	%	%
Expectations/perceptions	Considering data as assets	32.00%	51.60%	12.40%	3.90%	0.00%
	Increased opportunities for cooperation and participation within and outside organisations	40.50%	50.30%	7.80%	0.70%	0.70%
	Supporting organisational data infrastructures	35.30%	49.00%	13.10%	1.30%	1.30%
Challenges	Lack of training	28.80%	56.90%	11.80%	2.60%	0.00%
	Lack of technical tools	50.30%	39.20%	9.20%	1.30%	0.00%
	Organisational culture	26.20%	50.80%	20.00%	1.50%	1.50%

**Table 4:** Variance Inflation Factor (VIF).

Predictor Variable	VIF Value
Age Group	1.24
Education Level	1.18
Institutional Type	1.31
Years of Experience	1.42
Gender	1.09

All VIF values < 2.0 indicating no multicollinearity concerns.

**Table 5:** Regression Coefficients and Model 1 Statistics.

Predictor Variable	B	SE	Wald	*p*	OR	95% CI
(Constant)	−2.847	0.956	8.87	0.003	—	—
Postgraduate Education	1.842	0.617	8.90	0.003 **	6.31	1.89–21.1
Age 50–59 (vs. <30)	1.876	0.814	5.31	0.021 *	6.53	1.32–32.3
Age 40–49 (vs. <30)	1.243	0.698	3.17	0.075	3.46	0.88–13.6
Research Centre	1.127	0.587	3.68	0.055	3.09	0.98–9.76
Gender (Female)	0.315	0.487	0.42	0.518	1.37	0.53–3.56
Private Hospital	0.456	0.589	0.60	0.439	1.58	0.50–4.99

* *p* < 0.05; ** *p* < 0.01.

**Table 6:** Regression Coefficients and Model 2 Statistics.

**Predictor Variable**	**B**	**SE**	**Wald**	***p***	**OR**	**95% CI**
(Constant)	−3.152	1.041	9.16	0.002	—	—
Research Centre	1.845	0.671	7.55	0.006 **	6.33	1.69–23.7
Age 50–59 (vs. <30)	1.891	0.943	4.02	0.045 *	6.63	1.04–42.3
Years of Experience	0.068	0.035	3.81	0.051	1.07	1.00–1.14
Postgraduate Education	1.327	0.712	3.48	0.062	3.77	0.94–15.1
Private Hospital	0.521	0.725	0.52	0.473	1.68	0.41–6.94
Gender (Female)	0.289	0.578	0.25	0.617	1.33	0.43–4.13

* *p* < 0.05; ** *p* < 0.01.

**Table 7:** Participants in the focus group discussions.

Health Institutions	Participant ID	Role(s)	Background(s)	Years of Experience
Public hospital	P1	Head of the micro research unit	Cancer genetics	More than 20 years
	P2	Medical informatics specialist	Health informatics	More than 10 years
Private hospital	P3	Medical researcher	Genetic engineering and bioinformatics	More than 20 years
	P4	Lead—population health research	Genomics, research, data registry, public health bioinformatics	More than 20 years
Research Centre	P5	Head of the research centre	Genomic medicine and biobank unit	More than 20 years

**Table 8:** Status of FAIR implementation and data sharing in Saudi Arabia (survey and focus group discussions).

**Challenges/Obstacles**	**Potential Outcomes of Implementation**
1- Limited awareness. 2- Lack of training. 3- Inadequate technical and financial support. 4- Concerns regarding legal and ethical implications. 5- Lack of unified data governance framework.	1- Accelerate investment in the data infrastructures. 2- Lead to more efficient clinical outcomes and more informed decision-making for increasingly effective treatment. 3- Accelerate drug discovery, development of innovative medicines; enable collaboration. 4- Improve data interoperability and increase efficiency in data management. 5- Advance smart health technologies and accelerate the adoption of digital twin technology. 6- Promote the use of advanced AI tools to analyse health data.
**Actions needed to catalyse implementation in Saudi Arabia**	
1- Launch educational initiatives in data management practices. 2- Equip healthcare professionals with the skills necessary to adopt FAIR principles 3- Establish national standardised guidelines and regulations for health data sharing in alignment with FAIR principles. 4- Address the complexity of data ownership. 5- Ensure the privacy of patients and the confidentiality of their information. 6- Secure financial support and allocate adequate resources. 7- Encourage a culture of data sharing and open science practices.	

## Data Availability

The dataset produced and examined in this study can be accessed on 18 February 2025 OSF at the following URL: https://osf.io/gy38m/ with DOI: https://doi.org/10.17605/OSF.IO/GY38M. The repository contains: (1) Study Dataset (CSV format): 153 anonymized survey responses coded R001-R153; (2) Survey Instrument (PDF): Complete questionnaire in English with all validity metrics; (3) Qualitative Codebook (Excel): 47 codes with definitions, inclusion/exclusion criteria, and exemplar quotes; (4) Focus Group Transcripts (PDF): Three anonymized sessions totalling 18,500 words; (5) Statistical Analysis Scripts (SPSS): Complete syntax for all analyses; (6) Methodological Validation Report (PDF): Documentation of CVI, Cronbach’s alpha, and pilot testing. All materials are deposited under Creative Commons Attribution 4.0 license. Raw data files have been anonymized in accordance with ethical approval requirements (Approval No. HAPO-02-K-012-2024-02-2034). For questions regarding data contact: eaharbi@uqu.edu.sa.

## Abbreviation

FAIR	Findable, Accessible, Interoperable, Reusable
TAM	Technology Acceptance Model
CVI	Content Validity Index
SPSS	Statistical Package for the Social Sciences
OSF	Open Science Framework
GA4GH	Global Alliance for Genomics and Health
EOSC	European Open Science Cloud
